# Association between obesity-related dyspnea in daily living, lung function and body composition analyzed by DXA: a prospective study of 130 patients

**DOI:** 10.1186/s12890-022-01884-5

**Published:** 2022-03-25

**Authors:** Jean Hagenburg, Eric Bertin, Jean-Hugues Salmon, Aurore Thierry, Jeanne-Marie Perotin, Valérian Dormoy, Sandra Dury, Isabelle Gaubil, Lois Bolko, François Lebargy, Gaëtan Deslee, Claire Launois

**Affiliations:** 1 Service Des Maladies Respiratoires, CHU Reims, Reims, France; 2 Service d’Endocrinologie Diabète Nutrition, Centre Spécialisé Obésité, CHU Reims, Reims, France; 3 Service de Rhumatologie, CHU Reims, Reims, France; 4 Département de Méthodologie, CHU Reims, Reims, France; 5 INSERM UMR-S 1250 “Pathologies Pulmonaires Et Plasticité Cellulaire”, Reims, France

**Keywords:** Dyspnea, Obesity, Lung function, Body composition, Dual-energy X-ray absorptiometry

## Abstract

**Background:**

Obesity is a risk factor for dyspnea. However, investigations of daily living obesity-related dyspnea are limited and its mechanisms remain unclear. We conducted a cross-sectional study to analyze the relationships between dyspnea in daily living, lung function, and body composition in patients with obesity.

**Methods:**

One-hundred and thirty patients (103 women/27 men), candidate for bariatric surgery, with a mean ± SD Body Mass Index (BMI) of 44.8 ± 6.8 kg/m^2^ were included. Dyspnea was assessed by the modified Medical Research Council (mMRC) scale. Comorbidities, laboratory parameters, pulmonary function tests, arterial blood gases, six-minute walk test (6MWT), handgrip strength, and DXA body composition were analyzed.

**Results:**

Thirty-one percent of patients exhibited disabling dyspnea in daily living (mMRC ≥ 2). Compared with patients without disabling dyspnea (mMRC < 2), significant dyspnea (mMRC ≥ 2) was associated with a lower 6MWT distance (395 ± 103 m *vs* 457 ± 73 m, *p* < 0.001), lower lung volumes including Expiratory Reserve Volume (42 ± 28% *vs* 54 ± 27%, *p* = 0.024), Vital Capacity (95 ± 14 *vs* 106 ± 15%, p < 0.001) and Forced expiratory volume in one second (95 ± 13 *vs* 105 ± 15%, *p* = 0.002), a higher BMI (48.2 ± 7.7 *vs* 43.2 ± 5.7 kg/m^2^, *p* = 0.001) and a higher percentage of fat mass in the trunk (46 ± 5 *vs* 44 ± 5 *p* = 0.012) and android region (52 ± 4 vs 51 ± 4%, *p* = 0.024). There was no difference regarding comorbidities (except hypertension), laboratory parameters, and sarcopenia markers between patients with (mMRC ≥ 2) and without (mMRC < 2) disabling dyspnea.

**Conclusion:**

Dyspnea in patients with obesity is associated with a reduction in lung volumes and a higher percentage of fat mass in central body regions. How dyspnea and body composition may change with interventions like physical activity or bariatric surgery remains to be investigated.

**Supplementary Information:**

The online version contains supplementary material available at 10.1186/s12890-022-01884-5.

## Background

Although obesity is a significant risk factor for dyspnea [1–3], daily living obesity-related dyspnea has been the primary focus of a small number of studies [4–7]. While obesity has long been recognized as having significant effects on lung function [8], the mechanisms of obesity-related dyspnea remain unclear. In patients with obesity, lung volumes tend to be decreased [9, 10] and maximal inspiratory pressure (MIP) can also be low, reflecting decreased inspiratory muscle performance [11, 12]. Previous studies with relatively small population samples have shown that these abnormalities are moderately associated with daily living dyspnea in obese patients [4–7].

Associations between obesity and impaired skeletal muscle quality, poor physical performance, and a higher risk of sarcopenia have been well-established [13]. Dyspnea may also reduce physical activity as in chronic respiratory disease [14], subsequently leading to limb muscle impairment and deconditioning.

Body mass index (BMI) is the most commonly used measure to characterize obesity but is limited by its assessment of body weight relative to height, with no information on the body composition per se. Dual-energy X-ray absorptiometry (DXA) is a quick, weakly radiating, and reliable tool allowing to accurately measure body composition and to determine specific sites of fat deposition [15]. Regarding the effects of body fat distribution on lung function, DXA studies have shown that body fat deposition in the central regions (both thorax and abdomen) was associated with lung volume decrease [16]. These findings have been attributed to the restriction and load imposed by the excess fat mass on the thoracic cage and abdomen, placing the diaphragm, the main inspiratory muscle, into an inefficient position. Nevertheless, to our knowledge, the relationships between obesity-related dyspnea and body composition assessed by DXA have not been investigated so far.

The main objective of this study was to assess the relationships between dyspnea in daily living according to mMRC, lung function and body composition assessed by DXA in patients with obesity. We hypothesized that patients with disabling dyspnea would have a higher BMI and a higher proportion of fat mass, especially in the central regions of the body. The secondary objectives were to analyze the relationships between dyspnea in daily living and sarcopenia assessed by appendicular lean mass and handgrip strength measurements. We also investigated relationships between dyspnea, comorbidities, and laboratory parameters.

## Methods

### Patients and design

Between January 2017 to February 2020, consecutive adult patients with obesity referred to the Department of Nutrition at the University Hospital of Reims (France) for a project of bariatric surgery were systematically evaluated for dyspnea, lung function tests, handgrip strength, and body composition. All patients were included in the study before bariatric surgery, except those with a known respiratory, cardiac, or neuromuscular disease. Patients were also excluded if they did not perform the different investigations in a maximal period of two months.

Written information was provided, and each patient gave his written consent to participate. The study was approved by the Reims University Hospital Institutional Review Board (IRB-17–10-2012).

### Clinical characteristics and dyspnea assessment

Demographic data (age, sex), anthropometric characteristics (height, weight, BMI), medical comorbidities (hypertension, diabetes, dyslipidemia, severe obstructive sleep apnea syndrome (OSAS), defined as an Apnea–Hypopnea Index > 30/h), medical treatments (antihypertensive drugs, oral antidiabetics, insulin, cholesterol-lowering agents) and smoking status were systematically recorded.

The depression symptoms were assessed using the QD2A depression scale [17, 18]. This tool is a 13-item self-rated questionnaire of depressive symptomatology. Each item presents a statement that the subject answers as “true” or “false”, and the score is the number of items the subject marked “true” and varies from 0 to 13. A score of 7 or more indicates depression.

Dyspnea in daily living was evaluated using the mMRC scale [5, 19, 20]. This scale consists of five statements that almost entirely describe the range of dyspnea from none (grade 0) to almost complete incapacity (grade 4) (Table 1). The mMRC is the most commonly used validated scale to assess dyspnea in daily living in chronic respiratory diseases and a mMRC score ≥ 2 is considered as disabling dyspnea [19]. Borg > 3/10 is considered as dyspnea on exertion [21].

**Table 1:** Modified medical research council (mMRC) dyspnea scale

Grade of dyspnea	Description of breathlessness
0	I only get breathless with strenuous exercise
1	I get short of breath when hurrying on level ground or walking up a slight hill
2	On level ground, I walk slower than people of the same age because of breathlessness, or I have to stop for breath when walking at my own pace on the level
3	I stop for breath after walking about 100 yards or after a few minutes on level ground
4	I am too breathless to leave the house or I am breathless when dressing

### Muscle strength

Measurement of handgrip strength was conducted in a standardized manner by the use of a handgrip dynamometer type JAMAR (Saehan® Hydraulic Hand Dynamometer model SH 5001, Korea) in neutral rotation and adducted position of the shoulder, with the elbow flexed to 90 degrees, and forearm and wrist in a neutral position. Three trials from the dominant hand were measured in kilograms. The mean value was considered for the analyses. Grip strength less than 16 kg for women and less than 27 kg for men were considered low handgrip strength [22, 23].

### Lung function tests

#### Six-minute walk test

The six-minute walk test (6MWT) was performed in a 30-m long, flat, covered corridor, marked meter-by-meter, according to the American Thoracic Society guidelines [24]. Oxygen saturation and modified Borg scale subjectively assessing the degree of dyspnea graded from 0 to 10 were collected at the beginning and the end of the 6MWT. The distance covered was calculated at the end of the test.

#### Arterial blood gases

Arterial Blood Gases were measured in the morning in a sitting position on room air.

#### Pulmonary function testing

PFTs were performed according to the American Thoracic Society/European Respiratory Society guidelines [25] (BodyBox 5500 Medisoft Sorinnes, Belgium). Vital capacity (VC), forced vital capacity (FVC), forced expiratory volume in one second (FEV_1_), FEV_1_/FVC ratio, expiratory reserve volume (ERV) were measured during spirometry [26]. Residual volume (RV), functional residual capacity (FRC), and total lung capacity (TLC) were measured during plethysmography [27]. As the plethysmography cabin is not suitable for patients weighing more than 150 kg, spirometric measurements only (including FEV_1_, VC, FVC, FEV_1_/FVC, and ERV) were performed in patients weighing more than 150 kg. Results are expressed in milliliters and percentage of predicted values. A restrictive defect was defined as a TLC < 80% of predicted value. The carbon monoxide diffusing capacity of the lung (DLCO) was measured and expressed in percentage of predicted values [28]. As data concerning patients ethnicity was not collected in this study, ethnicity was not taken into account in PFTs predicted values results.

#### Evaluation of inspiratory and expiratory muscle strength

Respiratory muscle strength consisted of measuring Maximal Inspiratory Pressure (MIP) and Maximal Expiratory Pressure (MEP). The maximum value of three available tests that varied by less than 20% was recorded. Results were expressed in cmH2O and percentage of predicted values [29].

### Laboratory parameters

Hemoglobin (Hb), C reactive protein (CRP), and N-terminal prohormone of brain natriuretic peptide (NT-pro-BNP) were determined from a blood sample.

### Body composition assessment

Body composition was determined by DXA scan (Hologic Horizon™ DXA System QDR®, Vilvoorde, Belgium). DXA was quantified by the body tissue absorption of photons that were emitted at two energy levels to resolve body weight into bone mineral density (BMD), lean (LM), and fat (FM) soft tissue masses [30]. FM (kg), BMD + LM (kg), and FM percentage (%) for standard body regions, such as the trunk, lower limbs, android, and gynoid regions delineated by specific anatomical landmarks were analyzed. The trunk region was defined as the region horizontally below the chin, with vertical borders lateral to the ribs and oblique lines through the femoral neck. The lower limb region was defined as the region under the oblique lines through the femoral necks and within the leg lines. Android and gynoid regions were selected as regions of interest (ROI) using the software provided by the manufacturer. Briefly, the android ROI was defined as a portion of the abdomen included between the line joining the two superior iliac crests (lower boundary) and extending cranially up to 20% of the distance between this line and the chin. The gynoid ROI upper boundary was defined as 1.5 times the height of the android ROI below the iliac crest to a line equal to twice the height of the android ROI (lower boundary). As some patients (n = 45) exceeded the scan area dimensions concerning the arms, whole body, and upper limbs region data were not analyzed. Appendicular lean mass index (ALMI) was calculated as the sum of the lean mass of both the upper (when available) and the lower limbs adjusted for height (m^2^) (or as the sum of the lean mass of an upper limb in whole included in the scan multiplied by 2 and lower limbs adjusted for height (m^2^)) [31]. Sarcopenic obesity was defined according to *Baumgartner *et al*.* [32] as ALMI < 7.26 kg/m^2^ in men and 5.45 kg/m^2^ in women.

### Statistical analysis

It’s a pilot study with a prospective inclusion of patients over 3 years (no sample size calculation). Quantitative variables were described as mean ± standard deviation (SD) and qualitative variables as number and percentage. Patients were separated into two groups according to their mMRC dyspnea scale: mMRC < 2 (no disabling dyspnea in daily living) and mMRC ≥ 2 (disabling dyspnea in daily living). Variables associated with mMRC scale were studied using Student or Wilcoxon or Khi2 or Fisher exact tests according application’s conditions. A multivariate analysis using an ascending stepwise logistic regression models for dyspnea (mMRC ≥ 2 versus mMRC < 2) was peformed. Variables proposed were BMI, Borg > 3/10, predicted VC, predicted FVC, predicted FEV1, predicted ERV, predicted FRC and predicted TLC. Association between the trunk fat mass or the android fat mass percentage and pulmonary function testing were studied using the Pearson’s correlation coefficient. There was no imputation of missing data. A p-value < 0.05 was considered statistically significant. All analyses were performed using SAS version 9.4 (SAS Institute Inc., Cary, NC, USA).

## Results

### Patient characteristics

One-hundred and seventy-three consecutive patients were included in the study. Thirty-two of them were excluded because of an inability to perform PFTs and/or DXA and eleven because of an interval longer than two months between respiratory assessment and DXA. The data of the remaining 130 patients (103 women and 27 men) were analyzed. Of note, 111 patients (85%) performed respiratory assessment and DXA on the same day (Flowchat of study participants in Additional file 1).

Clinical, anthropometric, and demographic characteristics of the patients are presented in Table 2. The mean BMI was 44.8 ± 6.8 kg/m^2^ and the mean Android/Gynoid fat mass ratio was 1.09 ± 0.09.

**Table 2:** Clinical and demographic characteristics of the 130 obese patients

	Patients (n = 130)	Women (n = 103)	Men (n = 27)
Age (years)	42 (± 11)	42 (± 11)	42 (± 11)
Height (cm)	167 (± 8)	164 (± 6)	178 (± 6)
Weight (kg)	124.6 (± 22.2)	119.2 (± 19.5)	145.2 (± 20.2)
BMI (kg/m^2^)	44.8 (± 6.8)	44.4 (± 6.6)	46.0 (± 7.2)
Comorbidities			
Hypertension	44 (34%)	30 (29%)	14 (52%)
Diabetes	24 (18%)	16 (16%)	8 (30%)
Dyslipidemia	19 (15%)	11 (11%)	8 (30%)
Severe OSAS	36 (28%)	21 (21%)	15 (56%)
Treatments			
Antihypertensive drugs	38 (29%)	27 (26%)	11 (42%)
Oral antidiabetics	22 (17%)	15 (15%)	7 (26%)
Insulin	11 (8%)	7 (7%)	4 (15%)
Cholesterol-lowering agents	8 (6%)	5 (5%)	3 (12%)
Smoking history (n = 103)			
Current	13 (28%)	8 (28%)	5 (29%)
Former	33 (32%)	21 (27%)	12 (50%)
Never	56 (54%)	49 (62%)	7 (30%)
Pack-years	16 (± 15)	11 (± 10)	25 (± 20)

Data are expressed as mean (± SD) or number (%)

BMI: Body mass index; OSAS: Obstructive Sleep Apnea Syndrome

Overall, pulmonary function tests remained in the normal range for most of the patients except for ERV (577 ± 395 mL, 50 ± 28%). Regarding inspiratory and expiratory muscle strength, MIP was 61 ± 27 cmH_2_O (70 ± 31%) and MEP was 73 ± 36 cmH_2_O (66 ± 30%).

#### Dyspnea assessment

As shown in Table 3, 74% of the patients experienced dyspnea in daily living with a mMRC score > 0, and 31% disabling dyspnea with a mMRC score ≥ 2. Thirty-nine percent of patients described dyspnea on exertion (Borg ≥  > 3/10). There was no significant difference between men and women regarding dyspnea.

**Table 3:** Dyspnea assessment of the 130 obese patients

	Patients (n = 130)	Women (n = 103)	Men (n = 27)	*p*
mMRC scale (/4)				
mMRC ≥ 1	96 (74%)	76 (74%)	20 (74%)	1
mMRC ≥ 2	40 (31%)	29 (28%)	11 (41%)	0.244
Borg scale (/10) (n = 119)				
Borg at rest ≥ 1	13 (11%)	12 (13%)	1 (4%)	0.463
Borg after 6MWT ≥ 1	106 (89%)	84 (88%)	22 (92%)	1
Borg after 6MWT > 3	55 (46%)	44 (46%)	11 (46%)	0.97

Data are expressed as mean (± SD) or number (%)

mMRC: modified Medical Research Council; 6MWT: six-minute walk test

### *Comparisons of clinical parameters, lung function tests, and laboratory parameters between patients with mMRC* < *2 and patients with mMRC* ≥ *2*

There were no statistically significant differences between patients with (mMRC ≥ 2) and without (mMRC < 2) disabling dyspnea regarding treatments (23% *vs* 14%, *p* = 0.312 for oral antidiabetics; 13% *vs* 7%, *p* = 0.312 for insulin; 5% vs 7%, *p* = 1 for cholesterol-lowering agents; 38% *vs* 26%, p = 0.148 for antihypertensive drugs) and comorbidities (25% *vs* 16%, *p* = 0.225 for diabetes; 18% *vs* 13%, *p* = 0.594 for dyslipidemia; 36% *vs* 25%, *p* = 0.207 for severe OSAS) except for hypertension (48% *vs* 28%, *p* = 0.044). No significant difference was found regarding the depression state according to the QD2A depression scale (9% in patients with mMRC < 2 (n = 6) *vs* 11% in patients with mMRC ≥ 2 group (n = 3), *p* = 0.720). The percentage of active smokers (22% *vs* 56%, *p* = 0.092) and the number of pack-years (15 ± 14 *vs* 21 ± 22, *p* = 0.512) were similar in the two groups of patients.

Compared with patients with mMRC < 2, patients who experienced disabling dyspnea (mMRC ≥ 2) had a lower 6MWT distance and a significant reduction in lung volumes (Table 4). Two patients (2.2%) had a restrictive defect in the mMRC < 2 group and 1 patient (2.5%) in the mMRC ≥ 2 group. Multivariate analysis analysis using an ascending stepwise logistic regression models for dyspnea (mMRC ≥ 2 versus mMRC < 2) showed that only predicted FVC and Borg > 3/10 were significantly associated to the mMRC status (BMI, Borg > 3/10, predicted VC, predicted FVC, predicted FEV1, predicted ERV, predicted FRC and predicted TLC were proposed variables).

**Table 4:** Comparison of 6-min walk test, arterial blood gases, pulmonary function tests and laboratory parameters between patients with mMRC < 2 and patients with mMRC ≥ 2

	mMRC < 2 (n = 90)	mMRC ≥ 2 (n = 40)	*p*
6-min walk test (n = 119)			
6-min walk test distance (m)	457 (± 73)	395 (± 103)	0.0006**
6-min walk test distance (% pred)	88 (± 15)	83 (± 19)	**0.1517**
SpO2 at rest (%)	98 (± 1)	96 (± 6)	**0.073**
SpO2 after 6MWT (%)	94 (± 3)	94 (± 3)	0.577
Borg at rest (/10)	0.2 (± 0.4)	0.4 (± 0.9)	0.422
Borg after 6MWT (/10)	3.3 (± 2.1)	4.7 (± 2.7)	0.014*
Borg ≥ > 3 after 6MWT (/10)	32 (39%)	23 (64%)	0.01*
Arterial blood gases (n = 124)			
pH	7.42 (± 0.02)	7.41 (± 0.03)	0.312
PaO_2_ (mmHg)	93 (± 13)	94 (± 17)	0.663
PaCO_2_ (mmHg)	37 (± 3)	37 (± 4)	0.770
HCO_3_^−^ (mmol.L^−1^)	23 (± 2)	23 (± 2)	0.465
Pulmonary function tests			
Spirometry (n = 130)			
VC (mL)	3766 (± 847)	3362 (± 799)	0.005*
VC (% pred)	106 (± 15)	95 (± 14)	0.0007**
FVC (mL)	3729 (± 843)	3324 (± 812)	0.006*
FVC (% pred)	105 (± 15)	95 (± 13)	0.002*
FEV_1_ (mL)	3032 (± 657)	2646 (± 597)	0.0008**
FEV_1_ (% pred)	100 (± 15)	90 (± 15)	0.0004**
FEV_1_/FVC (% pred)	82 (± 6)	80 (± 7)	0.362
ERV (mL)	627 (± 404)	464 (± 353)	0.024*
ERV (% pred)	54 (± 27)	42 (± 28)	0.014*
Plethysmography (n = 110)			
RV (mL)	2069 (± 668)	1832 (± 710)	0.336
RV (% pred)	122 (± 33)	110 (± 36)	0.197
FRC (mL)	2719 (± 641)	2841 (± 739)	0.127
FRC (% pred)	97 (± 20)	88 (± 22)	0.0504*
TLC (mL)	5760 (± 1023)	5225 (± 1133)	0.033*
TLC (% pred)	109 (± 16)	99 (± 16)	0.0036*
DLCO (% pred)	90 (± 15)	85 (± 20)	0.166
Inspiratory muscle strength (n = 127)			
MIP (cmH_2_O)	63 (± 29)	56 (± 21)	0.358
MIP (% pred)	73 (± 33)	64 (± 25)	0.189
Expiratory muscle strength (n = 127)			
MEP (cmH_2_O)	73 (± 37)	74 (± 36)	0.888
MEP (% pred)	67 (± 30)	66 (± 30)	0.907
Laboratory parameters (n = 103)			
Hemoglobin (g/L)	135 (± 13)	132 (± 14)	0.245
CRP (mg/L)	8.5 (± 7.8)	10.7 (± 9.7)	0.193
NT-pro-BNP > 50 (pg/mL)	15 (22%)	8 (29%)	0.601

Data are expressed as mean (± SD) or number (%). *p < 0.05; **p < 0.001

p is in bold when it is < 0.05

mMRC: modified Medical Research Council; 6MWT: six-minute walk test; SpO2: Pulse Oxygen Saturation; PaO2: Partial arterial pressure of oxygen, PaCO2: Partial arterial pressure of carbon dioxide; HCO3-: bicarbonate anion; VC: Vital capacity; FVC: Forced vital capacity; FEV_1_: Forced expiratory volume in one second; ERV: Expiratory reserve volume; RV: Residual volume; FRC: Functional residual capacity; TLC: Total lung capacity; DLCO: Diffusing capacity of the lung for carbon monoxide; MIP: Maximal inspiratory pressure; MEP: Maximal expiratory pressure; CRP: C-reactive protein; NT-pro-BNP: N-terminal prohormone of brain natriuretic peptide

### *Comparison of body composition between patients with mMRC* < *2 and mMRC* ≥ *2*

Comparisons of body composition between patients without (mMRC < 2) and with disabling dyspnea (mMRC ≥ 2) are presented in Table 5. Patients with disabling dyspnea (mMRC ≥ 2) had a higher BMI, a higher mass, and a higher fat mass whatever the analyzed body segment (trunk, lower limbs, android, and gynoid regions) than patients with mMRC < 2. They also had a higher percentage of fat mass in the trunk and the android region than patients with mMRC < 2. There was no difference in sarcopenia markers (handgrip strength and appendicular lean mass index) between these 2 groups of patients. There were no significant correlation between the trunk fat mass or the android fat mass percentage with the following factors: VC, FVC, FEV1, ERV, TLC (in predicted values) (data not shown).

**Table 5:** Comparison of body composition between patients with mMRC < 2 and patients with mMRC ≥ 2

	mMRC < 2 (n = 90)	mMRC ≥ 2 (n = 40)	*p*
Height (cm)	167 (± 9)	166 (± 8)	0.723
Weight (kg)	120.9 (± 20.7)	133.0 (± 23.5)	0.0052*
BMI (kg/m^2^)	43.2 (± 5.7)	48.2 (± 7.7)	0.001*
Hand grip dynamometer (n = 112)			
Hand grip strength (kg)	27 (± 9)	28 (± 10)	0.853
Low hand grip strength ^Ŧ^	5 (7%)	3 (8%)	1
Dual X-ray Absorptiometry			
Low ALMI ^ŦŦ^ (n = 85)	0 (0%)	0 (0%)	1
Trunk			
Mass (kg)	55.58 (± 10.90)	61.33 (± 13.08)	0.017*
% total body mass	47 (± 5)	47 (± 4)	0.719
LM-BMD (kg)	31.16 (± 5.52)	32.69 (± 5.70)	0.117
FM (kg)	24.42 (± 6.39)	28.64 (± 8.79)	0.008*
FM (%)	44 (± 5)	46 (± 5)	0.012*
Lower limbs			
Mass (kg)	39.68 (± 7.10)	43.83 (± 7.93)	0.004*
% total body mass	33 (± 3)	33 (± 3)	0.970
LM-BMD (kg)	20.45 (± 4.23)	21.94 (± 4.56)	0.055
FM (kg)	19.22 (± 4.51)	21.89 (± 5.31)	0.014*
FM (%)	48 (± 6)	50 (± 6)	0.175
%FM Trunk/% FM Lower limbs	0.91 (± 0.13)	0.93 (± 13)	0.288
Android			
Mass (kg)	10.14 (± 2.34)	11.60 (± 2.56)	0.002*
% total body mass	8 (± 1)	9 (± 1)	0.021*
LM-BMD (kg)	4.97 (± 1.16)	5.49 (± 1.12)	0.012*
FM (kg)	5.17 (± 1.30)	6.12 (± 1.60)	0.001*
FM (%)	51 (± 4)	52 (± 4)	0.024*
Gynoid			
Mass (kg)	18.69 (± 3.46)	20.42 (± 3.52)	0.009*
% total body mass	16 (± 1)	16 (± 1)	0.713
LM-BMD (kg)	9.88 (± 1.97)	10.47 (± 2.03)	0.098
FM (kg)	8.81 (± 2.00)	9.95 (± 2.08)	0.009*
FM (%)	47 (± 5)	49 (± 5)	0.074
Android/gynoid fat mass ratio	1.09 (± 9)	1.09 (± 9)	0.821

Data are expressed as mean (± SD) or number (%)

*p < 0.05; **p < 0.001

p is in bold when it is < 0.05

mMRC: modified Medical Research Council; BMI: Body mass index; ALMI: Appendicular lean mass index; TM: Total mass; FM: Fat mass; LM: Lean mass; BMD: Bone mineral density

^Ŧ^: hand grip strength was considered low when < 16 kg in women and < 27 kg in men; ^ŦŦ^: ALMI was considered low when < 5.45 kg/m^2^ in women and < 7.26 kg/m^2^ in men

### Daily living dyspnea according to mMRC scale depending on BMI

**Fig. 1:**
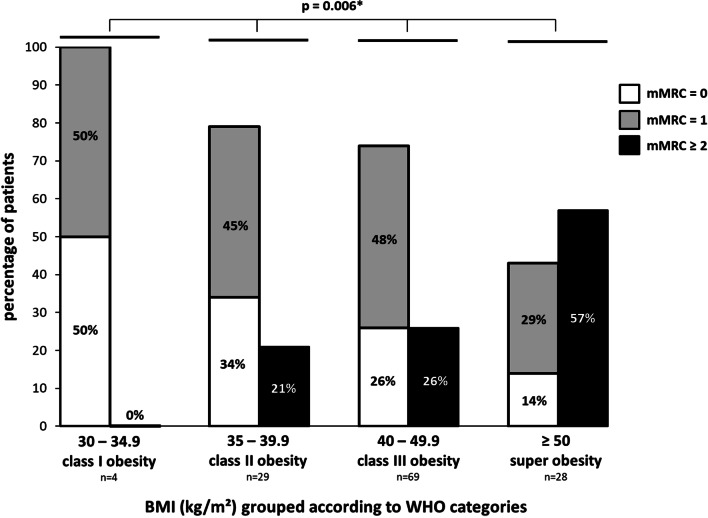
Daily living dyspnea according to mMRC scale depending on BMI grouped according to WHO categories. BMI: Body mass index; WHO: World Health Organization; mMRC: modified Medical Research Council. *: *p* < 0.05

Depending on BMI grouped according to World Health Organization categories [33, 34], the percentage of patients with mMRC 0, mMRC 1, and mMRC ≥ 2 was significantly different (*p* = 0.031) as well as the percentage of patients with mMRC < 2 and mMRC ≥ 2 (p = 0.006) (Fig. 1).

## Discussion

This study analyzed the relationships between dyspnea in daily living, lung function, and body composition assessed by DXA in obese patients and demonstrates that disabling dyspnea in daily living was associated with lower lung volumes and 6MWT distance, and a higher BMI and fat mass, especially in the central regions of the body.

In large cohorts, 80% of obese adults experience dyspnea after climbing two flights of stairs [35] and approximately one-third of obese adults report dyspnea when walking up a hill (1). Obese adults are also twice as likely as adults with normal BMI to have dyspnea mMRC score ≥ 2 [3]. The proportion of dyspnea in our cohort was similar to previous studies with about three-quarters of the patients experiencing dyspnea in daily living (mMRC ≥ 1) and more than a third describing disabling dyspnea in daily living (mMRC score ≥ 2, i.e. walk slower than people of the same age on level ground) and dyspnea on exertion (Borg > 3 after 6MWT) [21]. Interestingly, there was no difference in dyspnea severity between men and women in this study.

Dyspnea encompasses an array of unpleasant respiratory sensations that vary according to the underlying cause and patient characteristics. In this study, demographic characteristics, medical comorbidities except for hypertension, QD2A depression score, and smoking status were similar between patients with and without disabling dyspnea in daily living according to the mMRC dyspnea scale.

As expected, patients with disabling dyspnea in daily living (mMRC ≥ 2), who had also higher BMI and fat mass than patients with mMRC < 2, covered a lower distance during the 6MWT than patients with mMRC < 2 [5].

It is well known that obesity causes substantial changes to the mechanics of the lungs and chest wall that affect lung function. The most frequent abnormality associated with obesity is a decrease in ERV, which is exponentially correlated with increased BMI [9]. While obesity significantly reduces ERV and consequently FRC (FRC = ERV + RV), it has very little effect on VC and TLC [9]. RV is typically within the normal range in the presence of obesity. Other dynamic measures of lung function such as FEV_1_ and FVC are slightly reduced in people with obesity [36], but FEV_1_/FVC ratio is usually unaffected. We found similar results concerning lung function in this cohort of obese patients, showing that patients with disabling dyspnea in daily living (mMRC ≥ 2), who had also higher BMI, had a significant reduction in measures of lung function affected by obesity (VC, FVC, FEV1, ERV, FRC, TLC).

Effects of obesity on inspiratory and expiratory muscle strength are variable and inconsistent [11, 12, 37]. Respiratory muscle function might be impaired by a myopathy or by the load imposed on the diaphragm by obesity itself. Contrary to *Collet *et al. [4], we did not find a significant association between disabling dyspnea and inspiratory muscle strength. A possible explanation is that respiratory muscle strength is assessed by volitional methods and the patient’s motivation and effort can affect the results.

Despite the absence of consensus on the definition of sarcopenic obesity, it is commonly accepted as the combination of obesity and muscle impairment, either defined by low muscle mass and/or poor muscle strength/function. In a large cohort from the National Health and Nutrition Surveys, the prevalence of sarcopenic obesity is 17% in obese patients aged 60 to 70 years [38]. In our study, patients were younger with no patient exhibiting a low appendicular lean mass and very few patients with low handgrip strength. Furthermore, there was no association between these variables assessing muscle impairment and the presence of disabling dyspnea.

Disabling dyspnea according to mMRC was associated with an increase in weight, BMI, and fat mass in absolute value for all body segments. Interestingly, patients with disabling dyspnea also presented an increase in the percentage of fat mass for the central regions of the body: trunk and android region. *Sutherland *et al*.* also showed that both thoracic and abdominal body fat had an impact on lung volumes [16]. In our study, patients with disabling dyspnea had also lower lung volumes. Taken together, these data support the hypothesis that dyspnea may be mediated by the deposition of adipose tissue around the thorax restricting expansion, and/or by abdominal adiposity impeding diaphragmatic excursion.

Our results have several clinical implications. First, it provides clinicians with a glimpse of the dyspnea endured by obese patients especially in patients with high and very high BMI (Fig. 1). Second, there is a significant effect of adiposity on dyspnea and this relationship is robust regardless of the adiposity measurement (BMI, weight, fat mass in all analyzed body segments). Thus, assessing the effect of adiposity on dyspnea may be adequately undertaken using a simple measurement, such as BMI, in clinical practice.

One of the strengths of our study is the assessment of the relationships between dyspnea according to the mMRC scale, a very complete respiratory assessment (6MWT, arterial blood gases, PFTs, inspiratory and expiratory muscle strength), laboratory parameters, depression scale, and body composition assessed by DXA. As anxiety does not modify the mMRC impact score of dyspnea, it was not assessed in the study [39]. Our results highlight a significant association between the presence of disabling dyspnea, reduction in lung volumes, and increase in BMI and fat mass, especially in the central region of the body which is known to be associated with lung volume reduction. his study has several limitations. First, as data concerning patients ethnicity was not collected in this study, ethnicity was not taken into account in PFTs predicted values results. Nevertheless, we believe that the vast majority of included patients were caucasien and consequently that this missing data didn’t modify PFTs predicted values results. Second,, it was conducted in a single center, which may limit the generalizability of the results. Third, the study cohort included only candidates for bariatric surgery (predominantly women, relatively young) and consequently does not reflect the whole population of obese individuals. Moreover, our study does not provide information regarding the effects of interventions like bariatric surgery. It has been shown that bariatric surgery improves dyspnea in about two-thirds of patients [6]. As body composition significantly changes after bariatric surgery with reduced whole-body and regional fat mass and especially decreased percentage of android fat mass [40], it would be interesting to study the relationships between body composition modification and dyspnea improvement after bariatric surgery.

## Conclusion

This prospective study showed that dyspnea in daily living in obese patients is associated with a reduction in lung volumes and higher BMI, possibly related to a higher percentage of fat mass in central body regions. It remains to be investigated how dyspnea and body composition may change with interventions like physical activity or bariatric surgery.

## Supplementary Information

**Additional file 1.** Flowchart of study participants.

## Data Availability

The datasets during and/or analysed during the current study available from the corresponding author on reasonable request.

## Abbrevations

6MWT: Six-minute walk test
ALMI: Appendicular lean mass index
ATS: American Thoracic Society
BMD: Bone mineral density
BMI: Body mass index
CRP: C-reactive protein
DLCO: Carbon monoxide diffusing capacity of the lung
DXA: Dual-energy X-ray Absorptiometry
ERV: Expiratory reserve volume
FEV1: Forced expiratory volume in one second
FM: Fat mass
FRC: Functional residual capacity
FVC: Forced vital capacity
Hb: Hemoglobin
LM: Lean mass
MEP: Maximal expiratory pressure
MIP: Maximal inspiratory pressure
mMRC: Modified Medical Research Council dyspnea scale
NT-pro-BNP: N-terminal prohormone of brain natriuretic peptide
OSAS: Obstructive sleep apnea syndrome
PFTs: Pulmonary function tests
ROI: Regions of interest
