# Supplementary material for: Association between obesity-related dyspnea in daily living, lung function and body composition analyzed by DXA: a prospective study of 130 patients
Source: BMC Pulm Med. 2022 Mar 25;22:103. doi: 10.1186/s12890-022-01884-5 (PMC8957162; doi:10.1186/s12890-022-01884-5)
Supplement: Supplementary file 1 — Additional file 1. Flowchart of study participants. [file 12890_2022_1884_MOESM1_ESM.pptx]

## Slide 1
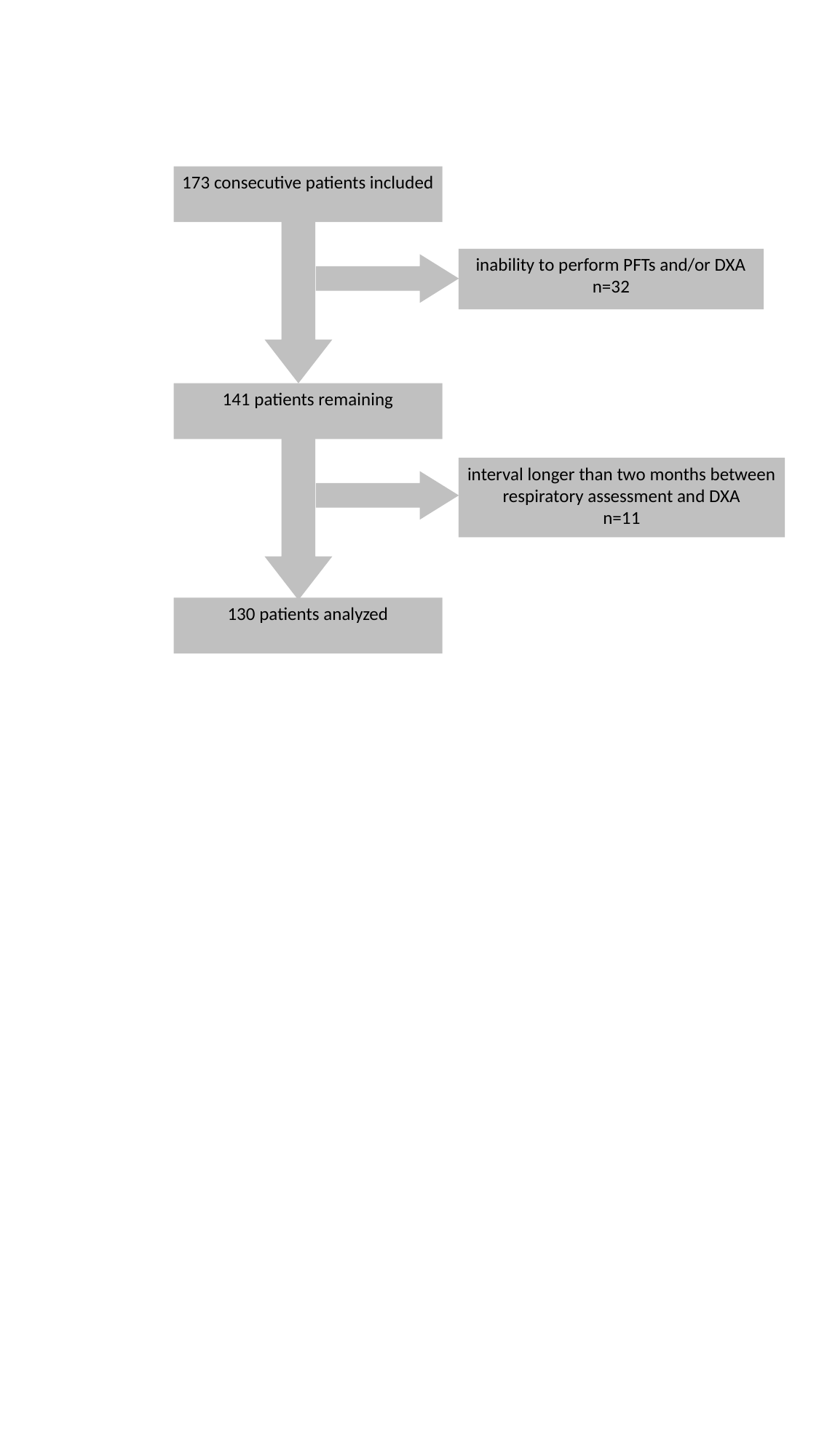

173 consecutive patients included
inability to perform PFTs and/or DXA
n=32
141 patients remaining
interval longer than two months between respiratory assessment and DXA
n=11
130 patients analyzed
